# Multifaceted Roles of Mitochondrial Components and Metabolites in Metabolic Diseases and Cancer

**DOI:** 10.3390/ijms21124405

**Published:** 2020-06-20

**Authors:** Jean Nakhle, Anne-Marie Rodriguez, Marie-Luce Vignais

**Affiliations:** 1 Institute for Regenerative Medicine & Biotherapy (IRMB), INSERM, Univ Montpellier, F-34090 Montpellier, France; jean.nakhle@umontpellier.fr; 2 Institute of Molecular Genetics of Montpellier (IGMM), CNRS, Univ Montpellier, F-34090 Montpellier, France; 3 Univ Paris Est Creteil, INSERM, IMRB, F-94010 Creteil, France; 4 EnvA, IMRB, F-94700 Maisons-Alfort, France; 5 EFS, Mondor Institute for Biomedical Research (IMRB), F-94010 Creteil, France; 6 AP-HP, Hopital Mondor, Service d’histologie, F-94010 Creteil, France

**Keywords:** mitochondria, electron transport chain (ETC), tricarboxylic acid (TCA) cycle, mitochondrial DNA (mtDNA), metabolism, mitochondria exchange, microbiota, metabolites, cancer, therapy

## Abstract

Mitochondria are essential cellular components that ensure physiological metabolic functions. They provide energy in the form of adenosine triphosphate (ATP) through the electron transport chain (ETC). They also constitute a metabolic hub in which metabolites are used and processed, notably through the tricarboxylic acid (TCA) cycle. These newly generated metabolites have the capacity to feed other cellular metabolic pathways; modify cellular functions; and, ultimately, generate specific phenotypes. Mitochondria also provide intracellular signaling cues through reactive oxygen species (ROS) production. As expected with such a central cellular role, mitochondrial dysfunctions have been linked to many different diseases. The origins of some of these diseases could be pinpointed to specific mutations in both mitochondrial- and nuclear-encoded genes. In addition to their impressive intracellular tasks, mitochondria also provide intercellular signaling as they can be exchanged between cells, with resulting effects ranging from repair of damaged cells to strengthened progression and chemo-resistance of cancer cells. Several therapeutic options can now be envisioned to rescue mitochondria-defective cells. They include gene therapy for both mitochondrial and nuclear defective genes. Transferring exogenous mitochondria to target cells is also a whole new area of investigation. Finally, supplementing targeted metabolites, possibly through microbiota transplantation, appears as another therapeutic approach full of promises.

## 1. Introduction

Mitochondria are descendants from alpha-proteobacteria that survived after their endocytosis by eukaryotic progenitors, more than 1.5 billion years ago. These bacterial ancestors became symbiotic with their host cells and gradually gave rise to the permanent organelles found today in almost all eukaryotic cells [1]. As a vestige of their bacterial endosymbiotic origin, mitochondria have retained their double membranes and circular genome [2]. However, this mitochondrial DNA (mtDNA) has been dramatically reduced over evolution, rendering mitochondria dependent on the nucleus for the expression of the vast majority of their proteins [2]. As a result, the biogenesis and functionality of mitochondria are tightly regulated by the continuous crosstalk with the nucleus [3]. The function usually assigned to mitochondria is their capacity to produce high amounts of ATP, from glucose breakdown, through oxidative phosphorylation (OXPHOS). OXPHOS-dependent ATP synthesis is supported by the mitochondrial electron transport chain (ETC), composed of protein complexes and organic molecules, which conveys electrons to molecular oxygen and creates the electrochemical gradient needed for the functioning of ATP synthase [4]. In tight connection with the ETC, metabolites are produced in the mitochondria through the tricarboxylic acid (TCA) cycle. The TCA cycle is composed of eight enzymes catalyzing the chemical breakdown of carbohydrates, fats, and proteins to produce either ATP or building blocks for the synthesis of nucleic acids, amino-acids, and lipids.

Beyond their role as energy and building-block producers, mitochondria also act as signaling organelles that govern cell fate by regulating essential biological processes, such as cell growth, differentiation, and apoptosis, as well as Ca^2+^ and redox homeostasis. Mitochondria are thus implicated in essential physiological and pathophysiological processes including tissue healing, inflammation, and cancer [5,6,7,8]. The complexity and diversity of mitochondrial functions are reflected by their wide-ranging proteome of more than 1000 proteins [9,10]. The already-extensive panel of mitochondrial functions has been further increased following the recent discovery of their capacity to translocate to other cells and to alter their behavior [11,12]. This signaling role of mitochondria is also supported by their recently-documented capacity to release metabolites as well as mitochondrial fragments, also called damage-associated molecular patterns (DAMPs) [13,14,15].

The severity of the diseases caused by mitochondrial dysfunctions confirms the critical importance of mitochondria for living cells. These diseases can originate from genetic mutations directly affecting the ETC activity or other mitochondrial functions. However, they can also be owing to unbalanced production of metabolites and mitochondrial components. The challenging identification of mitochondrial dysfunctions will need to be overcome in order to develop therapeutic strategies for the related metabolic, inflammatory, and malignant pathologies [14,16,17,18].

Here, we present an overview of recent findings on the critical involvement of mitochondrial metabolites/compounds in the signaling functions of mitochondria and on the emergence of novel therapies for diseases caused by mitochondrial dysfunctions.

## 2. Role of the Mitochondrial ETC and OXPHOS in Physiology and Disease

**Figure 1:**
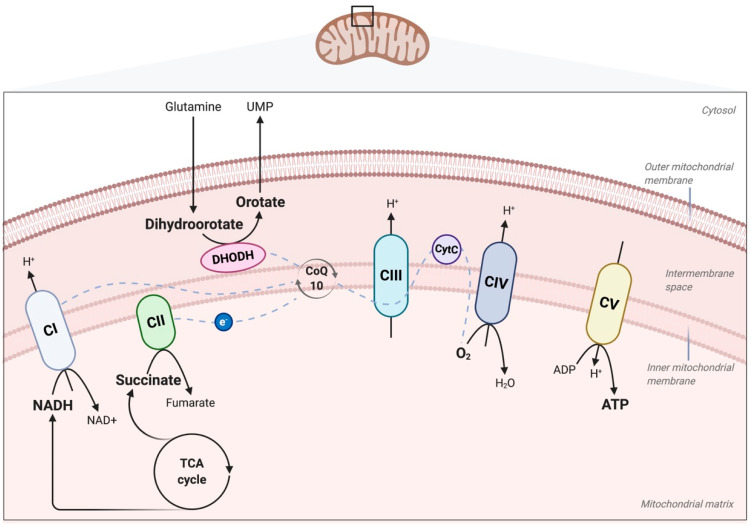
Oxidative phosphorylation (OXPHOS) through the electron transport chain (ETC) and adenosine triphosphate (ATP) production. OXPHOS is the process leading to ATP synthesis through the transport of electrons released by the oxidation of nicotinamide adenine dinucleotide (NADH) and succinate generated by the tricarboxylic acid (TCA) cycle. The transport of electrons is mediated by the ETC located in the mitochondrial inner membrane. The ETC is composed of the four complexes NADH–ubiquinone oxidoreductase (complex I), succinate–CoQ oxidoreductase (complex II), ubiquinol–cytochrome c oxidoreductase (complex III), and cytochrome c oxidase (complex IV), and of the free-electron carriers ubiquinone (CoQ10) and cytochrome c (CytC). Electrons are transferred through the ETC and finally to oxygen (O_2_) (dashed lines). This electron transfer is accompanied by a flow of protons (H^+^) from the mitochondrial matrix into the intermembrane space, across complexes I, III, and IV. The generated transmembrane electrochemical proton gradient allows the ATP synthase (complex V) to produce ATP. Also shown is the dihydroorotate dehydrogenase (DHODH), which participates in the electron transfer process through the oxidation of dihydroorotate to orotate, leading to de novo pyrimidine synthesis.

The respiratory machinery responsible for OXPHOS is located in the inner mitochondrial membrane. It includes the four complexes (I–IV) of the electron transport chain (ETC), the free-electron carriers ubiquinone and cytochrome c, and the ATP synthase (complex V) that produces OXPHOS-dependent ATP (Figure 1). The ETC machinery is encoded by both the nuclear DNA (nDNA) (80 proteins) and mitochondrial DNA (mtDNA) (13 proteins). Nuclear-encoded proteins (roughly 70% of total mitochondrial proteins) also have functions in ETC supercomplex (or respirasome) assembly [19] and in mtDNA replication and repair [20]. Therefore, nuclear and mitochondrial gene expressions need to be tightly coordinated to ensure effective mitochondrial functioning. Alterations in either result in mitochondrial defects, which makes their treatment particularly challenging. Mitochondrial DNA exhibits high mutation rates owing to (1) direct exposure to ETC-derived reactive oxygen species (ROS), (2) asymmetric replication leading to higher exposure of the single H-strand, (3) intramitochondrial dNTP pool imbalance favoring dGTP incorporation, and (4) low-efficiency DNA repair mechanisms [21]. Mitochondrial DNA mutations range from point mutations to large-scale DNA rearrangements. They can be found in cells together with their wild-type counterpart, a feature called heteroplasmy. Exceeding the critical heteroplasmy thresholds causes mutant mtDNA to trigger mitochondria-related pathologies [22].

Mitochondrial diseases are characterized by genetic alterations that primarily compromise oxidative phosphorylation (OXPHOS) and OXPHOS-dependent ATP generation. Mitochondrial diseases thus affect organs that mainly rely on OXPHOS for energy supply, including the eye, ear, liver, kidney, heart, and skeletal muscles, as well as the brain. These diseases are associated with syndromes such as CPEO (chronic progressive external ophthalmoplegia), KSS (Kearns–Sayre syndrome), LHON (Leber hereditary optic neuropathy), MIDD (maternally inherited diabetes and deafness), MELAS (mitochondrial encephalomyopathy with lactic acidosis and stroke-like episodes), mitochondrial encephalomyopathy (Leigh syndrome), MERRF (myoclonic epilepsy with ragged-red fibers), and NARP (syndrome neuropathy, ataxia, and retinitis pigmentosa). Mitochondrial disorders have also been associated with cancers in different organs [17,22,23], as detailed below.

## 3. Effects of Mutations in the Subunits of Complexes I and II of the ETC in Cancer

Mitochondrial disorders play a pivotal role in tumorigenesis by orchestrating metabolic reprogramming, apoptosis, and ROS signaling at virtually all stages, from tumor initiation to metastasis, and mtDNA mutations have been proposed as drivers/modifiers of tumorigenesis in many cancers [24,25,26]. Sequencing of mtDNAs from over 2000 tumor samples across over 30 tumor types revealed important features of the mitochondrial mutational signature [27,28]. These large-scale studies identified intrinsic replicative errors, characterized by an asymmetric bias towards C > T and T > C transitions on the mtDNA heavy and light strands respectively, as the most prominent source of mtDNA mutations, overpowering the 4% caused by ROS exposure. While neutral missense mutations were found to gradually drift towards homoplasmy, deleterious frameshift or nonsense mutations, often resulting in truncated proteins, were almost exclusively heteroplasmic, thus highlighting a selective pressure in cancer cells to retain residual mitochondrial functionality [27,28].

### 3.1. Complex I Mutation-Induced ROS Production Promotes Tumorigenesis through Phosphatidylinositol 3-Kinase (PI3K)/Protein Kinase B (AKT) Signaling, Hypoxia-Inducible Factor 1 Alpha (HIF1α) Stabilization, and NADPH-Oxidase 1 (NOX1) Signaling

Complex I of the ETC transfers electrons from NADH produced in the TCA cycle and from the β-oxidation of fatty acids to ubiquinone. This reaction is accompanied by the translocation of four protons from the mitochondrial matrix into the intermembrane space, which generates a transmembrane electrochemical gradient and drives final ATP production by complex V (Figure 1). Complex I is the largest (1 MDa) and most elaborate complex of the ETC. X-ray crystallography revealed a structure with two L-shaped arms: a peripheral arm that catalyzes the redox reaction and an inner membrane-embedded arm containing the proton-translocating machinery. Complex I is composed of 14 conserved core subunits, encoded by both the mitochondrial and nuclear DNAs, and of at least 30 additional nuclear-encoded accessory subunits. Half the core subunits (ND1, ND2, ND3, ND4, ND4L, ND5, ND6) are mitochondria-encoded and constitute the membrane-embedded arm, while the other half (NDUFS1, NDUFV1, NDUFV2, NDUFS2, NDUFS3, NDUFS8, NDUFS7) are nuclear-encoded and constitute the peripheral arm [19,29,30]. Complex I is the main target of the currently-identified mtDNA mutations. Taking into account both mtDNA and nDNA origins, mutations are found in all 14 catalytic core subunits, in 13 accessory subunits, and in at least 11 assembly factors [31]. They have been described in cancers [32] as diverse as head and neck [33,34], breast [27,35,36], thyroid [37,38,39], prostate [40], renal [39,41], and hepatocellular cancers [42]. The technique of transmitochondrial cybrids, which consists of repopulating ρ0 cells (depleted of their mtDNA) with exogenous mitochondria, has been widely used to establish the role of mtDNA variants in pathologies, independent of nDNA backgrounds [43]. Transmitochondrial cybrid studies thus showed that complex I mutants can exhibit both pro- and anti-tumorigenic effects in cancer, in an OXPHOS- and ROS-dependent fashion, as detailed below.

#### 3.1.1. PI3K/AKT Signaling

**Figure 2:**
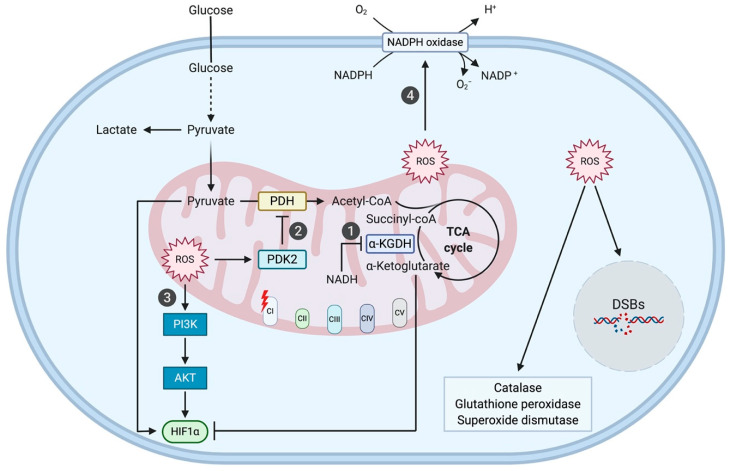
Effects of complex I mutations. (**1**) As a result of complete loss of complex I, NADH inhibits the TCA cycle enzyme α-ketoglutarate dehydrogenase (αKGDH). This increases α-ketoglutarate levels and results in HIF1α inhibition. (**2**–**4**) Complex I mutations increase reactive oxygen species (ROS) production. (**2**) ROS activate pyruvate dehydrogenase kinase (PDK2), which inhibits pyruvate dehydrogenase (PDH). Pyruvate accumulation activates HIF1α. (**3**) ROS activate the phosphatidylinositol 3-kinase (PI3K)/protein kinase B (AKT)/hypoxia-inducible factor 1 alpha (HIF1α) signaling pathway. (**4**) Mitochondrial ROS activate the NADPH oxidase, which produces cytoplasmic superoxide. ROS lead to genomic instability via double-strand breaks (DSBs) and activate the cellular anti-oxidant responses.

The ND5 m.12418insA frameshift mutation, found in colorectal cancers, leads to a truncated ND5 protein, which destabilizes the assembly of the membrane-embedded arm of complex I [44,45]. As shown in 143B osteosarcoma transmitochondrial cybrids, the effects of this mutant highly depended on its heteroplasmy level. While a 72% ND5 mutant heteroplasmy still retained 46% residual complex I activity, ND5 mutant homoplasmy (96%) led to loss of complex I function [44]. These effects were associated, as expected, with a gradual decrease of OXPHOS-dependent oxygen consumption and ATP production [46]. Heteroplasmic cybrids exhibited increased levels of mitochondria-specific ROS (but not of intracellular ROS), which correlated with the overexpression of the catalase, the glutathione peroxidase 4 (Gpx4), and the Cu-Zn superoxide dismutase (SOD1) anti-oxidative enzymes. This enhanced ROS production also activated the PI3K/AKT signaling pathway and the expression of downstream genes encoding the hypoxia-inducible factor 1 alpha (HIF1α) and the anti-apoptotic proteins B-cell lymphoma-extra large (BCL-XL) and induced myeloid leukemia cell differentiation protein (MCL1) (Figure 2) [47]. Osteosarcoma cybrids harboring a heteroplasmic ND5 mutation were thus endowed with resistance to oxidative stress and, subsequently, enhanced tumorigenicity upon subcutaneous injection in nude mice [46,47]. ND5-mutant homoplasmic cybrids, however, showed increased levels in both mitochondrial and intracellular ROS, which led to apoptosis and prevented tumor formation in vivo [46].

#### 3.1.2. HIF1α Stabilization

The ND1m.3571insC frameshift mutation, observed in thyroid oncocytic carcinoma, also generates a truncated ND1 protein, which leads to complex I disassembly [48]. Transmitochondrial osteosarcoma cybrids expressing this ND1 mutant totally lost their complex I activity and their basal oxygen consumption, revealing a dramatic OXPHOS deficit [49]. Complex I dysfunction also induced the accumulation of NADH, an allosteric inhibitor of α-ketoglutarate dehydrogenase, which led to increased α-ketoglutarate levels and to HIF1α destabilization (Figure 2). These osteosarcoma cybrids did not form tumors in vivo [49,50]. Another ND1mutation (missense m.3460G>A, A52T) showed a weaker phenotype as homoplasmic osteosarcoma cybrids retained 50% of complex I activity, with only a partial reduction in basal oxygen consumption [49]. These mutants were still able to form tumors upon xenograft in mice, a tumorigenic potential linked to cytoplasmic ROS accumulation and HIF1α stabilization [49]. A third type of mutation (m.4776G>A; A103T), found in the ND2 subunit of complex I in head and neck squamous cell carcinoma, also illustrates the role of ROS accumulation and HIF1α stabilization in tumorigenesis [33,34]. This mutation, which impaired cell respiration and increased ROS production, led to a feedback metabolic signaling involving pyruvate dehydrogenase (PDH) and pyruvate dehydrogenase kinase 2 (PDK2) [34]. ROS-induced PDK2 activity resulted in increased pyruvate, HIF1α activation and tumor growth (Figure 2) [34]. Finally, the highly-metastatic capacities of murine Lewis lung carcinoma and fibrosarcoma were attributed to the missense m.13997G>A (P25L) and frameshift m.13885insC mutations, respectively, in the mtDNA ND6-encoding gene, as further confirmed in cybrids [51]. The increased metastatic capacity of these complex I mutants was also associated with a higher ROS production, which led to high levels of HIF1α, of the anti-apoptotic MCL-1 protein, and of the vascular endothelial growth factor (VEGF), thus nurturing neoangiogenesis. Consistent with this role of ROS, pretreatment of highly-metastatic cybrids with the ROS scavenger *N*-acetylcysteine (NAC) decreased MCL-1 expression in vitro and reduced metastasis in vivo [51].

#### 3.1.3. NOX1 Signaling

NADPH-oxidase 1 (NOX1) activity was also shown to contribute to tumorigenesis, together with increased mitochondrial ROS, as a result of complex I dysfunction [52]. The various osteosarcoma cybrids analyzed, carrying the m.3460G>A/MT-ND1 (A52T), m.11778G>A/MT-ND4 (R340H), and m.14484T>C/MT-ND6 (M64V) point mutations, respectively, exhibited decreased OXPHOS and ATP production. Their enhanced tumorigenicity, upon xenograft in nude mice, was associated with increased levels of both mitochondrial and cytoplasmic ROS as well as increased activity of NOX1, resulting in the production of superoxide and hydrogen peroxide, major sources of cytoplasmic ROS (Figure 2) [52]. Overall, mutations in complex I appear to exert anti-tumorigenic effects only upon complete abolishment of complex I function, which prevents ROS production. Milder mutations, which preserve some complex I activity, enhance ROS production and stimulate tumor growth [46,49,52].

### 3.2. OXPHOS-Harmful Complex I Mutations Elicit Metabolic Compensation

#### 3.2.1. Shift towards Glycolysis

Several studies reported that OXPHOS dysfunction, as a consequence of complex I impairment, leads to a metabolic shift towards glycolysis, which enables cells to maintain cell growth and ATP production [33,34,46,49,52,53]. This metabolic shift was reported in transmitochondrial osteosarcoma cybrids harboring the m.12418insA/MT-ND5 mutation. Increasing the mutational load from 72% of mutant ND5 in heteroplasmic cybrids to 96% in homoplasmic cybrids gradually decreased respiration (from 53% to 17% of wild type (WT)) and OXPHOS-linked ATP production (from 21% to 2% of WT), while it concomitantly increased glucose uptake and lactate production (from 28% to 56% of WT) [46]. Likewise, as shown for another subunit of complex I, osteosarcoma cells harboring the severe m.3571insC/MT-ND1 mutation showed increased glucose uptake and lactate production, while the milder m.3460G>A/MT-ND1 mutant had a glucose metabolism similar to that of the wild-type cells [49]. This glycolytic shift observed in transmitochondrial osteosarcoma cybrids was ascribed to HIF1α activation [50]. HIF1α stimulated the expression of the glucose transporters 1 (SLC2A1) and 3 (SLC2A3) as well as a series of glycolytic enzymes, including phosphofructokinase (PFKP), glyceraldehyde 3-phosphate dehydrogenase (GAPDH), phosphoglycerate kinase (PGK1), and lactate dehydrogenase (LDHA) [50]. Taken together, these data indicate that complex I mutations could induce a Warburg-like effect in cancer cells, fostering tumor growth.

#### 3.2.2. Shift towards Complex II-Dependent Succinate Oxidation

Beyond the Warburg effect, a metabolic shift towards complex II-dependent succinate oxidation was recently described as an adaptive mechanism to compensate for OXPHOS dysfunction in high-grade prostate cancers, mutant for the ND1 subunit of complex I [54]. As a response to the inhibition of glutamate and malate oxidation and to the subsequent electron transport loss across complex I, ND1-mutant cancer cells were found to produce ATP by aerobic respiration through complex II-mediated succinate oxidation [54]. This metabolic reprogramming was associated with shorter patient survival, highlighting the critical role of complex II and succinate in malignancies associated with complex I dysfunction [54].

#### 3.2.3. Shift towards Serine Catabolism

The catabolism of serine, a precursor for nucleic acids, proteins, and fatty acids [55,56], was also shown to maintain OXPHOS activity in colon cancer cells with mutant complex I, by producing NADH and feeding it to the ETC [57]. Higher NADH levels than expected could be reached through serine catabolism because of the insensitivity to NADH concentrations of MTHFD2 (methylenetetrahydrofolate dehydrogenase 2), the enzyme involved in this conversion [57]. Overall, these findings demonstrate the various ways tumor cells manage to get around complex I dysfunction to maintain efficient OXPHOS.

#### 3.2.4. Complex I Loss Alleviates Complex V Dysfunction

By applying a genome-wide CRISPR screening on human K562 chronic myeloid leukemia cells concomitantly treated with oligomycin, an inhibitor of complex V, Mootha and colleagues identified a series of both synthetic lethal mutants and suppressors showing that complex I mutations can alleviate complex V dysfunction [58]. Mechanistically, concomitant inhibition of complexes I and V was accompanied by an increased reductive carboxylation of α-ketoglutarate [58]. Experimental depletion of cytosolic NADPH, known to drive reductive pathways, suppressed the protective effect of complex I loss against complex V inactivation, thus supporting the role of reductive metabolism in these effects [58].

### 3.3. Complex II Dysfunction Induces Tumorigenicity via Succinate Accumulation

Complex II, also known as succinate dehydrogenase (SDH) and succinate ubiquinone oxidoreductase, has the unique property of linking both the TCA cycle and the ETC by coupling succinate oxidation to fumarate within the TCA cycle with ubiquinone (coenzyme Q10) reduction to ubiquinol in the ETC. Complex II is composed of four subunits solely encoded by nuclear genes (SDHA, SDHB, SDHC, and SDHD). SDHA oxidizes succinate to fumarate, while reducing FAD to FADH2. Electrons from FADH2 are then transferred sequentially to SDHB and to ubiquinone at the inner membrane-embedded site formed by SDHC and SDHD. Contrary to complexes I, III, and IV, complex II-mediated electron transport is not accompanied by proton translocation into the mitochondrial intermembrane space [19,59].

Deleterious mutations in any of the four subunits of complex II decrease SDH activity and result in abnormal accumulation of succinate, as observed in complex II-mutated cells in vitro and in the extracellular fluids (plasma, urine, saliva, and feces) of complex II-deficient patients. Complex II mutations, principally affecting SDHB and SDHD subunits, have been associated with various cancers including hereditary paraganglioma and pheochromocytoma (i.e., neuroendocrine tumors of the paraganglionic tissue), gastrointestinal stromal tumors [60], and renal cell carcinoma [61]. Therefore, SDH has now been defined as a tumor suppressor and succinate as an oncometabolite [62,63].

#### 3.3.1. Complex II Mutations Inhibit 2-Oxoglutarate-Dependent Dioxygenases

**Figure 3:**
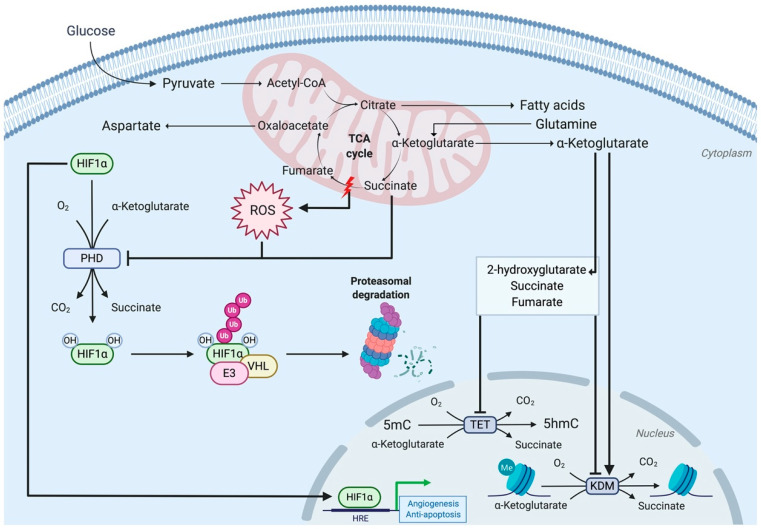
Role of TCA cycle intermediates in HIF1α stabilization and nuclear epigenetic modifications. Following its hydroxylation by prolyl-hydroxylases (PHDs), HIF1α is recognized by the von Hippel–Lindau (VHL) complex that targets it for proteasomal degradation. The inactivation of complex II of the ETC leads to ROS accumulation and inhibition of PHD activity by succinate, preventing HIF1α hydroxylation and degradation. Upon translocation to the nucleus, HIF1α binds to the hypoxic response element (HRE) and activates gene transcription, contributing to tumorigenesis. Complex II-deficient cells primarily rely on glutamine to fuel the TCA cycle and utilize oxaloacetate to generate aspartate for their biosynthetic pathways. The TCA cycle also produces metabolites that act as epigenetic modifiers. The α-ketoglutarate is a cofactor of 2-oxoglutarate-dependent dioxygenases, including the ten-eleven translocation (TET) family of DNA demethylases and the histone lysine demethylase (KDM) family. The metabolites 2-hydroxyglutarate, succinate, and fumarate, which are structurally similar to α-ketoglutarate, act as antagonists of TET- and KDM-catalyzed reactions.

The R22X nonsense SDHD mutation found in hereditary paraganglioma and pheochromocytoma generates a truncated SDHD protein of 21 amino acids (instead of 159), resulting in the loss of complex II electron transfer and enzymatic activities and in the activation of the HIF1α signaling pathway [64,65]. The R22X nonsense SDHD mutation leads to succinate accumulation, which inhibits the activity of prolyl hydroxylase (PHD) and, consequently, induces HIF1α stabilization [65]. PHD is a 2-oxoglutarate (2-OG, also known as α-ketoglutarate)-dependent dioxygenase that, in normal settings, hydroxylates HIF1α and leads to its poly-ubiquitylation by the Von Hippel–Lindau protein (pVHL) complex and, ultimately, to its degradation by proteasomes [66] (Figure 3). Succinate is structurally similar to 2-OG. It thus acts as a competitive inhibitor for PHD and promotes HIF1α stabilization [67,68].

Succinate accumulation resulting from complex II mutations was shown to inhibit other 2-OG-dependent dioxygenases, including the Jumonji C (JmjC)-domain containing histone lysine demethylases (KDMs) [69] and the ten-eleven translocation (TET) family of DNA hydroxylases [70] (Figure 3). TET enzymes mediate DNA oxidative demethylation through 5-methylcytosine (5mC) hydroxylation into 5-hydroxy-methylcytosine (5hmC) [70,71]. Succinate accumulation was shown to reduce TET-induced levels of 5hmC in human embryonic kidney cells (HEK293T) with SDHA/B knockdown as well as in mice livers with transient SDHA knockdown [72]. In line with these observations, DNA hypermethylation was found in SDHx-mutated paraganglioma and pheochromocytoma samples and in SDH-deficient gastrointestinal stromal samples, as a result of succinate inhibition of KDM and TET enzymes [73,74,75].

#### 3.3.2. The Hypermethylator Phenotype: A Double-Edged Sword for Complex II-Mutated Cancers

As mentioned above, SDH mutations can promote tumorigenesis by a succinate-mediated genome-wide epigenetic remodeling [74,76]. As shown in mouse SDHB-knockout chromaffin cells, increased succinate levels were associated with CpG island hypermethylation in the promoters of genes encoding proteins such as the matrix-metalloprotease inhibitor SPOCK2, the metastasis suppressor DNAJA4, and the cell adhesion marker KRT19 [74], thus resulting in a derepression of epithelial-to-mesenchymal transition (EMT). Succinate accumulation following SDHB-knockdown also led to an EMT-like phenotype in a murine serous ovarian carcinoma cell line, which was attributed to H3K27 hypermethylation [77]. The now-established succinate-mediated “hypermethylator” phenotype was also recently shown to synergize with HIF2α in establishing a mesenchymal-like phenotype and enhancing the metastatic potential of SDHB-knockout mouse chromaffin cells in vivo [75].

Yet, the hypermethylator phenotype of succinate accumulation was also found to sensitize complex II-deficient cancer cells to chemotherapy, as shown in paragangliomas and pheochromocytomas following treatment with the alkylating agent temozolomide [78]. This was attributed to the decreased expression of the DNA repair enzyme O^6^-methylguanine-DNA methyltransferase (MGMT) consequent to its promoter hypermethylation, as detected in patient SDHB-mutated paraganglioma and pheochromocytoma metastasis [78,79]. Similar inhibition of DNA repair processes was reported for SDHB-deficient YUNK1 kidney cell lines as a result of histone lysine demethylase (KDM) inhibition [80]. Deficiency in homologous recombination rendered SDHB-knockdown cells sensitive to synthetic lethality with poly(ADP)-ribose polymerase (PARP) inhibitor drugs olaparib and BMN-673, as shown both in vitro and in vivo, thus suggesting potential therapies for renal cell cancer (HLRCC) [80]. Taken together, these studies indicate that the succinate-induced hypermethylator phenotype acts as a double-edged sword in the physiopathology of cancers harboring SDH mutations, as it can either promote or inhibit cancer progression, depending on the genes targeted for methylation.

### 3.4. Metabolic Compensation Following Complex II Mutations Affecting OXPHOS

#### 3.4.1. Preferential Usage of Glucose and Glutamine

As complex II links the ETC to the TCA cycle, alterations of its functions expectedly result in important metabolic rewiring, found to support the bioenergetic needs of tumors. In particular, murine SDHB-deficient serous ovarian carcinoma cells were shown to exhibit an unbalanced TCA cycle, with low production of fumarate and malate, owing to high succinate levels. In addition, these complex II-deficient cells preferentially used glucose for anaerobic ATP production (through glycolysis) and glutamate for fueling the TCA cycle [77]. Similar preferential incorporation of glutamine carbons in the TCA cycle has been reported in murine SDHB-knockout chromaffin cells [81]. These data suggest that SDH-deficient cells rewire their central carbon metabolism through differential metabolite usage, with glucose as a source of ATP production and glutamine as a major fuel for the TCA cycle.

#### 3.4.2. Dependence on Pyruvate Carboxylation

In addition to its effects on glucose and glutamine metabolism, complex II deficiency leads to alterations in the metabolism of aspartate, a major precursor for non-essential amino acid, protein, and nucleotide biosynthesis. Seminal studies elegantly demonstrated that SDH-deficient cells use oxaloacetate, generated by pyruvate carboxylation, to produce aspartate, while wild-type cells rely on acetyl-CoA originating from pyruvate oxidation. These results suggest that SDH-deficient cells switch from pyruvate oxidation to carboxylation to sustain their aspartate anabolic needs. These data were further confirmed by the growth arrest of SDH-deficient cells following pyruvate carboxylation inhibition [81,82]. Taken together, these results confirm previous studies on the role of the ETC for cell proliferation, owing to its support of the synthesis of aspartate [83,84].

#### 3.4.3. CII_low_, an Energy Consumption Regulator

An interesting study by the Neuzil team recently identified an alternative assembly of complex II, termed CII_low_, and composed only of the SDHA subunit. This CII_low_ complex, detected in patients with SDHB-mutated paragangliomas, was linked to poor survival [62]. Expression of CII_low_ complex in SDHB-deficient cancer cells was associated with a decrease in de novo pyrimidine synthesis and cell proliferation along with an upregulation of catabolic and salvage pathways. The expression of such a CII_low_ complex may thus result from the metabolic adaptation of SDHB-deficient cancer cells, which are less proliferative, but endowed with high invasive and metastatic capacities [62].

## 4. Role of Complex III of the ETC for Physiological Functions

### 4.1. Links between Complex III Activity in T_reg_ Cells and Immune Function

As developed above, the diverse complexes of the electron transport chain (ETC) have an essential role for the effective coupling of mitochondrial respiration and energy production. Nonetheless, the defective activity of ETC complexes can have further functional consequences that depend on mitochondria-produced metabolites and reach beyond mere mitochondrial activity. T regulatory (T_reg_) cells are a subset of CD^4+^ T cells characterized by a mitochondrial metabolism. Chandel and collaborators recently showed that the activity of complex III (ubiquinol-cytochrome c reductase) of the ETC is needed for the immune suppressive function of T_reg_ cells and that its ablation leads to fatal inflammatory disease in treated mice, through modified metabolite production [85]. Mice whose T_reg_ cells are deficient in complex III were generated, by deleting the gene encoding the Rieske iron-sulfur protein (RISP), one of its essential subunits. Although T_reg_ cell proliferation and survival, as well as Foxp3 expression, were maintained in these mice, their immune suppressive capacity was lost. Increased concentrations of the 2-hydroxyglutarate (2-HG) and succinate metabolites, which are known inhibitors of the TET-family of DNA demethylases (Figure 3), were found responsible for the DNA hypermethylation phenotype, for the altered gene expression in T_reg_ cells and, eventually, for the disruption of their immune suppressive function [85].

### 4.2. Links between Complex III Activity in Haematopoietic Stem Cells and Haematopoiesis

Haematopoietic stem cells (HSCs) display a mainly glycolytic phenotype. However, the importance of HSC mitochondrial activity for hematopoiesis was demonstrated by the same team, by depleting complex III in murine fetal hematopoietic stem cells. As for RISP-null T_reg_ cells, RISP-null fetal HSCs maintained their proliferation. However, HSC impaired respiration was accompanied by a decreased NAD^+^/NADH ratio and, as observed for RISP-null T_reg_ cells, levels of the metabolites 2-hydroxyglutarate and succinate were increased, as that of fumarate. The functional consequences of RISP-dependent complex III depletion and the resulting unbalanced metabolite production were the inability of HSCs to generate multipotent progenitors, leading to mice anemia and fetal death [86].

### 4.3. Links between Complex III Activity in Endothelial Cells and Angiogenesis

Endothelial cells (ECs) also primarily use glycolysis for ATP production, vessel sprouting, and angiogenesis. However, in addition to glucose that fuels glycolysis, endothelial cells can also take up fatty acids and glutamine that fuel the TCA cycle and, further, the ETC. By antimycin inhibition of complex III in human umbilical vein endothelial cells (HUVECs) in vitro, Chandel and collaborators showed that complex III is necessary for angiogenesis through the maintenance of NAD^+^/NADH ratios, aspartate concentrations, and proliferation of endothelial cells. These effects of a dysfunctional complex III in reducing NAD^+^/NADH ratios, proliferation, and angiogenesis were confirmed in vivo in mice harboring a deletion of the *Uqcrq* gene, encoding the ubiquinol-binding protein QPC, a critical subunit of complex III. They were accompanied by a loss of postnatal retinal and lung angiogenesis, as well as melanoma angiogenesis in a B16-F10 melanoma model [87].

These studies highlighted the physiological consequences of a dysfunctional complex III of the mitochondrial ETC, for immunity, hematopoiesis, or angiogenesis. Some of these effects were linked to the overproduction of metabolites like 2-hydroxyglutarate and succinate, or fumarate, which interestingly appeared to be cell-type dependent, suggesting other levels of regulation.

## 5. Versatile Roles of Mitochondrial Components in Physiology and Disease

### 5.1. The Role of Ubiquinone (Coenzyme Q10), Activated by the Mevalonate Pathway, in Cancer

**Figure 4:**
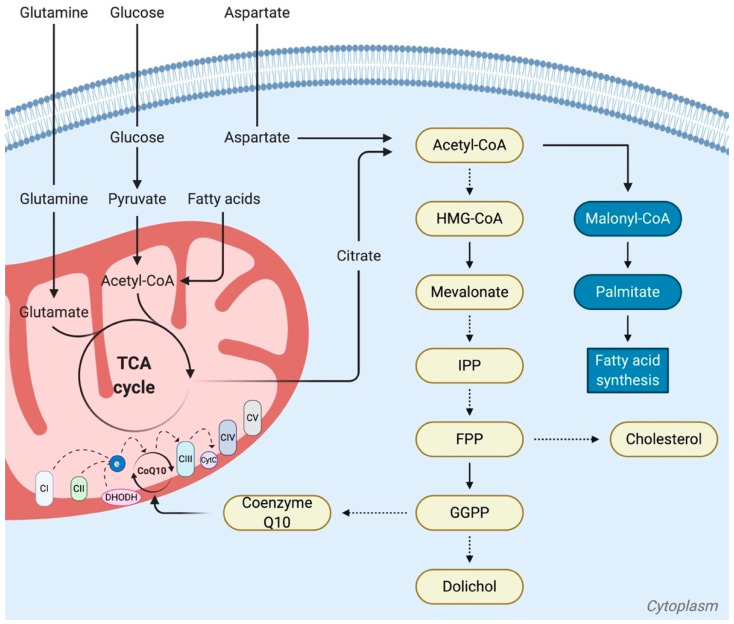
The mevalonate and the fatty acid synthesis pathways. Acetyl-CoA, derived from glucose, glutamine, or citrate following its export to the cytosol, is converted through the mevalonate pathway into several metabolites including cholesterol and coenzyme Q10. Coenzyme Q10 transfers electrons from complexes I and II of the electron transport chain, as well as from dihydroorotate dehydrogenase (DHODH), to complex III. Acetyl-coA also acts a precursor for fatty acid synthesis, through its conversion to malonyl-CoA, and then to palmitate. The mevalonate pathway is represented in yellow boxes. The fatty acid synthesis pathway is represented in blue boxes. Dashed arrows represent multiple steps. HMG-CoA, 3-hydroxy-3-methylglutaryl CoA; IPP, isopentenyl-diphosphate; FPP, farnesyl diphosphate; GGPP, geranylgeranyl-diphosphate; TCA cycle, tricarboxylic acid cycle.

Ubiquinone, also known as coenzyme Q10 (CoQ10), is an important electron carrier located in the inner mitochondrial membrane, where it transfers electrons from complexes I and II to complex III of the electron transport chain (ETC) (Figure 1). Ubiquinone is thus involved in the regulation of oxidative stress and ROS production. Ubiquinone is also a downstream metabolite of the mevalonate pathway. The mevalonate pathway uses acetyl-CoA, derived from glucose, glutamine, and/or acetate metabolism, to produce mevalonate; farnesyl-pyrophosphate (FPP); and, thereafter, different metabolites including cholesterol and ubiquinone [88] (Figure 4). The mevalonate pathway is often upregulated in cancers, which leads to increased mitochondrial concentrations of CoQ10. Statin inhibition of the mevalonate pathway is beneficial and statin treatment has been correlated with tumor cell apoptosis and reduced mortality in diverse cancers, notably breast cancer, pancreatic adenocarcinoma, and hepatocellular carcinoma [88]. As shown for pancreatic ductal adenocarcinoma (PDAC) tumor cells, ubiquinone levels are lowered by statin treatment, resulting in increased oxidative stress and ROS production. However, this oxidative stress triggers redox-active metabolic pathways aimed at lowering ROS levels, including the increased import of cystine for downstream glutathione production [89]. Therefore, the dysfunctional role of ubiquinone in the mitochondria of PDAC cells can be addressed by the concomitant targeting of the upstream mevalonate pathway (with statins) and of the metabolic glutathione-based compensation of excessive ROS production (with cystine/glutamate xCT antiporter inhibitors). As demonstrated in PDAC murine models, this effective approach triggers cancer cell death while sparing the mitochondrial functions [89].

### 5.2. Changing Dogmas about the Mitochondrial Role of CPT1, in both Synthesis and Oxidation of Fatty Acids

Lipids are important metabolites for membrane building and, therefore, for cell proliferation. They also provide cellular energy, act as signaling entities, and are involved in intercellular communication. All these functions allow lipid metabolism to contribute to cancer progression [90]. Both activation of fatty acid (FA) synthesis and FA oxidation have been linked to cancer progression. As recently reviewed [90], enzymes involved in fatty acid β-oxidation were overexpressed in diverse cancers and their inhibition was shown to curb cancer progression. Such is the case for CPT1 (carnitine palmitoyltransferase 1), a protein associated with the outer mitochondrial membrane, allowing the transport of long-chain fatty acids into the mitochondrial matrix. CPT1-dependent transfer of long-chain acyl groups from coenzyme A to carnitine constitutes the rate-limiting enzymatic process for the oxidative degradation of fatty acids [91].

However, CPT1 is now demonstrated to affect cancer cell proliferation by mechanisms relying on anabolic FA synthesis rather than FA β-oxidation (FAO) [92]. This novel role for CPT1 was uncovered by changing the tools to inhibit CPT1 activity. Instead of the etomoxir-dependent pharmacological inhibition of CPT1, *CPT1* knock downs were performed [92]. Studying the effects of CPT1 using high concentrations of etomoxir was marred by its off-target inhibition of complex I of the electron transport chain, a feature that was reported for the BT549 breast cancer cell line [92] as well as for T cells [93,94,95]. *CPT1* knock down in the BT549 breast cancer cell line demonstrated the role of CPT1 for cell proliferation, independently of FAO. CPT1 was actually found necessary for mitochondrial morphology maintenance, regulated mitochondrial lipid levels, polarized mitochondrial membrane, and efficient respiratory chain coupling [92]. As suggested by the authors, an important CPT1 function may thus be to provide long-chain fatty acids for anabolic processes in the mitochondria, needed for healthy cells and exploited in enhanced cancer cell proliferation [92].

### 5.3. Different Types of Mitochondria Linked to Lipid Metabolism

Attributing both antagonistic functions of FA synthesis and FA oxidation to mitochondria might be counter-intuitive. However, the recently-reported existence of metabolically-distinct mitochondrial subpopulations—one endowed with FA oxidation activity, the other involved in lipid droplet formation—could reconcile these two different mitochondrial activities [96,97]. A mitochondrial population associated with lipid droplets was characterized in brown adipose tissue (BAT). These peridroplet mitochondria (PDM) showed a high bioenergetic capacity that supported triacylglyceride (TAG) synthesis and lipid droplet formation. The protein composition of these mitochondria and the structure of their cristae appeared different from that of cytosolic mitochondria. They remained as an isolated pool different from cytosolic mitochondria owing to their low fusion-fission activity. In BAT cells isolated from cold-exposed mice, PDM abundance was reduced two fold in association with enhanced β-oxidation activity [97]. Mitochondria recruitment to lipid droplets is not limited to BAT, as also reported in mouse embryonic fibroblasts (MEFs) and in striated muscle upon nutrient deprivation, as reviewed by Benador et al. [96]. Lipid droplet accumulation has been associated with therapy resistance for various cancers, including glioma, colon, kidney, lung, and prostate cancers [98]. Whether PDM represent a population of mitochondria that can be targeted therapeutically in these pathologies will be worth investigating.

## 6. Mitochondria as Signaling Organelles: Functional Effects of Mitochondrial Exchange between Cells

Mitochondria are known to act as signaling organelles owing to their production of reactive oxygen species, structural compounds, and metabolites. As described above, metabolites produced by the TCA cycle can be used for the biosynthesis of macromolecules such as lipids, proteins, and nucleotides, as well as for epigenome modifications and post-translational protein changes [99]. As a result, mitochondria regulate a wide range of biological functions, including survival, growth, and differentiation.

However, the effects of mitochondria are not restricted to the cells that originally produced them, as we now know that, quite unexpectedly, mitochondria can translocate between cells. By their capacity to get transferred between cells, mitochondria can thus provide signaling cues to other cells. Numerous studies have now documented the diverse effects generated by intercellular mitochondria transfers and shown that they depend on the physiological/pathophysiological conditions and, more specifically, on the state of both mitochondria-provider and -recipient cells. The parts below provide an overview of the functional effects of intercellular mitochondria transfers in tissue repair, inflammation, and cancer progression, with a focus on the role of metabolites and structural compounds generated in the mitochondria-recipient cells.

### 6.1. Biological Effects of Intercellular Mitochondria Transfers

The past two decades revealed that mitochondria constantly communicate with both the cytosol and the nucleus under normal and stress conditions, thus eliciting adaptive cellular biological responses. More recently, the concept that mitochondria could exert biological effects outside from their original cells emerged from the observation that whole mitochondria, or parts of mitochondria, can translocate from one cell to the other. One example of intercellular mitochondrial communication is provided by the crosstalk of dying cells, following tissue injury, with the innate immune system of recipient cells, through the release of mitochondrial molecules including mitochondrial DNA (mtDNA), N-formyl peptides, ATP, or cardiolipin [100,101]. These mitochondrial products, recognized as damage-associated molecular patterns (DAMPs) by specific receptors on innate immune cells, are capable of directly activating the innate immune system and triggering adaptive inflammatory responses [102,103]. In addition to the crosstalk mediated by mitochondrial fragments, cells have been shown to communicate with each other by exchanging whole mitochondria.

Intercellular transfers of whole mitochondria have been reported to occur both in vitro and in vivo, and in both physiological and pathological conditions. This phenomenon is involved in several biological processes such as tissue repair, inflammation, and cancer progression (see reviews [11,12,104,105]). Mitochondria can translocate from one cell to the other by different modalities. These include thin membrane channels called tunneling nanotubes (TNTs) that ensure the connection and the mitochondria transfer between mitochondria-donor and -acceptor cells [11,12,104,105]. Mitochondria can also be transferred through their release in the extracellular space, either encapsulated inside microvesicles or as free organelles [104,106,107]. Importantly, the biological effects promoted by intercellular mitochondria transfers are highly dependent on the state of both mitochondria-donor and mitochondria-recipient cells. The outcome of these transfers can be beneficial, for instance, in tissue healing; on the other hand, they can also enhance tumor progression [11,12,104,105].

The vast majority of the studies addressing the process of intercellular mitochondria transfers have been conducted on mesenchymal stem cells (MSCs) as donor or acceptor cells for the conveyed mitochondria. These studies revealed that, compared with other cells, MSCs demonstrate a high capacity to donate their mitochondria to neighboring cells. This capacity is attributed to high expression of Miro-1, an outer mitochondrial membrane Rho-GTPase that mediates mitochondria trafficking through TNTs [108,109,110]. Mitochondria donation from MSCs to damaged or cancer cells has invariably been shown to improve recipient cell survival (see reviews [11,12,104,105]). Therefore, this process has important consequences in tissue regeneration and in cancer progression and aggressiveness (resistance to chemotherapy). MSCs have also been reported to transfer mitochondria to immune cells including T cells and macrophages. In these settings, transferred mitochondria decreased inflammation by promoting recipient immune cell differentiation towards an anti-inflammatory phenotype [111,112,113,114,115,116]. On the contrary, the transfer to T cells of mitochondria originating from myeloid cells was shown to exacerbate inflammation and to lead to asthma aggravation [117]. In addition, mitochondria transfer from pro-inflammatory monocytes to endothelial cells was associated with vessel inflammation observed in cardiovascular diseases [118]. It is worth mentioning that intercellular mitochondria transfers can be bidirectional, eliciting distinct biological responses in the two communicating cells [119]. For instance, and as mentioned above, MSCs can transfer mitochondria to damaged cells, which results in improved survival. However, MSCs can also acquire mitochondria released from damaged cells. These mitochondria function as signaling organelles that alert MSCs of a danger situation and trigger an adaptive wound-healing response [119].

The presence in the bloodstream of free or membrane-encapsulated respiratory-competent mitochondria has also been recently reported. Although the biological impact of these circulating mitochondria has yet to be determined, a role in regulating systemic inflammation through their interactions with immune cells could be speculated [107,120,121]. This pro-inflammatory role of circulating mitochondria is supported by observations made in organs from deceased patients and used for allotransplantation. In particular, circulating whole mitochondria and mtDNA levels were strongly correlated with the inflammation initiated by neutrophil activation and early allograft dysfunction, as shown for liver transplantation [120]. Besides, the concentration of free mitochondria in the blood of cancer patients correlated with metastasis, suggesting that circulating mitochondria are involved in cancer cell-to-cell communication processes [107].

### 6.2. OXPHOS Induced by Mitochondria Transfer in Tissue Repair and Cancer

The first discovered biological effect assigned to intercellular mitochondria transfers was their capacity to improve the survival of recipient cells, either damaged following a stress injury [122,123] or, for cancer cells, treated by chemotherapy [124]. Increased recipient cell survival was invariably correlated with enhanced OXPHOS activity and ATP production. The most convincing proof of the importance of functional OXPHOS restoration in the mitochondria-recipient cells was provided by studies using, as either mitochondria-donor or -recipient cells, ρ0 cells, which are devoid of mtDNA and have an impaired respiratory chain. The pioneering work of Prockop and collaborators thus demonstrated the OXPHOS activity restoration in ρ0 A549 lung adenocarcinoma cells owing to mitochondria transfer from cocultivated MSCs [125]. Likewise, Neuzil and collaborators found that mitochondria transfer occurred in vivo, from host stroma cells to engrafted ρ0 melanoma or ρ0 breast cancer cells, and that this process fully restored the respiration in the acceptor ρ0 cancer cells [126,127].

Beside the studies conducted with ρ0 cells, many works reported that mitochondria are transferred during cocultures from MSCs to damaged cells, including cardiomyocytes [122,128], endothelial cells [129], bronchial epithelial cells [130], corneal epithelial cells [131], and neuronal cells [132], or with leukemic, bladder, and breast cancer cells [133,134,135]. In addition, the physiological relevance of this process was strengthened by the in vivo demonstration of an increase in OXPHOS activity and ATP production following mitochondria transfer from mouse lung alveolar epithelial cells injured through exposure to LPS [123], to rotenone [110], or to cigarette smoke [130].

Finally, the prerequisite for transferred mitochondria to harbor functional respiration in order to exert their cytoprotective effects was reinforced by using ρ0 MSCs, which were unable to protect damaged cells against apoptosis [122].

### 6.3. Metabolic Reprogramming of MSCs and Cancer Cells by Metabolites Supplied by Mitochondra Transfer

As mentioned above, the acquisition of functional mitochondria leads to the enhancement of OXPHOS activity and ATP production in mitochondria-recipient cells. This process is of critical importance in damaged and dying cells owing to the role of ATP in restoring cellular bioenergetics. Beyond its impact on ATP levels, OXPHOS activation may also reflect an activation of the TCA cycle with an increased generation of some metabolites, with the OXPHOS and the TCA cycle being tightly coordinated [14,99]. It is still poorly documented to what extent intercellular mitochondria transfers contribute to the metabolic reprogramming of recipient cells through enhanced production of TCA cycle metabolites. However, two recent works demonstrated that TCA cycle metabolites are involved in the signaling mediated by the transferred mitochondria in recipient cancer cells and MSCs. As shown by Neuzil’s group, ρ0 melanoma and breast cancer cells lacking functional OXPHOS were unable to form tumors in mice. However, acquisition of respiratory-competent mitochondria from stromal cells allowed these ρ0 malignant cells to become highly proliferative, leading to tumor formation and progression [127]. Mechanistically, the authors demonstrated that OXPHOS-mediated ATP production was not required for tumor progression, but that, instead, OXPHOS function was critical for the generation of orotate, which is an essential intermediate for pyrimidine de novo synthesis [136,137]. In particular, OXPHOS restoration in ρ0 cells was found to re-activate the enzymatic activity of dihydroorotate dehydrogenase (DHODH), which is the ubiquinone-oxidoreductase responsible for orotate formation through dihydroorotate oxidation (Figure 1) [136,138]. As pyrimidine serves as building blocks for DNA replication and transcription, this explains why restoration of OXPHOS in ρ0 cells is essential to sustain cell proliferation [137]. In the absence of a functional respiratory chain, as is the case in ρ0 cells prior to intercellular mitochondria transfers, DHODH is unable to oxidize dihydroorotate, thus blocking pyrimidine synthesis and, as a result, arresting DNA replication and cell division [136].

Another example of the role of TCA cycle metabolites in mediating the effects of exogenous mitochondria is provided by recent observations from our laboratory indicating that the respiratory-competent mitochondria released by activated platelets can be engulfed by MSCs and can stimulate the MSC pro-angiogenic activity [139]. Citrate levels were increased in MSCs following platelet mitochondria transfer. Citrate was identified as a key metabolite initiating metabolic remodeling and functional alterations in recipient MSCs. Citrate was shown to stimulate recipient MSCs through its export to the cytosol where it fueled the ATP citrate lyase enzyme (ACLY), leading to the activation of fatty acid synthesis (Figure 4) and, subsequently, to the stimulation of the angiogenic activity of MSCs [139].

### 6.4. Role of DAMPs, Generated by the Transfer of Damaged Mitochondria, in Regulating Inflammation and MSC Activation

Following tissue injury, damaged cells have been reported to release their mitochondria to the extracellular environment [119,140,141]. As discussed below, the translocation of these damaged mitochondria to target cells, such as immune cells or reparative progenitors cells, functions as signaling cues, able to alert the rest of the body of a danger situation [140,142,143]. In contrast to the above-mentioned studies where transferred mitochondria needed to be fully functional to behave as pro-survival factors or as metabolite suppliers, mitochondria transferred from suffering cells mediate their effects by the means of mitochondrial fragments or DAMPs generated by recipient cells. Several DAMPs produced by recipient cells following the intercellular transfer of damaged mitochondria have been involved in inflammation [106,141]. One example is provided by the role of mitochondria released by activated platelets to neutrophils in the stimulation of the innate immune system [106]. Following their engulfment by neutrophils, platelet-derived mitochondria have been shown to have their membranes hydrolyzed by the phospholipase A2 enzyme, this mitochondria degradation leading to the production of various DAMPs, including lysophospholipids, fatty acids, and mtDNA, which are known to trigger leukocyte activation to have their membranes hydrolyzed by the phospholipase A2 enzyme [106].

More recently, Zhu and colleagues reported that both apoptotic and necrotic cells released whole mitochondria in the extracellular compartment [141]. Strikingly, the authors observed that the mitochondria released by the two types of dying cells did not support the same systemic effects following their internalization in cultivated macrophages, with mitochondria from apoptotic cells generating more inflammation than those derived from necrotic cells. Mechanistically, cardiolipin was identified, in the recipient macrophages, as the DAMP responsible for the activation of the inflammasome, a multi-protein complex able to detect danger signals and to trigger the secretion of the pro-inflammatory IL-1α and IL-18 cytokines [141]. How mitochondrial cardiolipin exerts its pro-inflammatory effects in these settings remains to be formally demonstrated. One possible explanation suggested by the authors is that the mitochondrial cardiolipin was externalized from the inner to the outer mitochondrial membrane in the apoptotic mitochondria. This conformational change would allow the binding of cardiolipin to the inflammasome sensor NLRP3 (NOD-, LRR-, and pyrin domain-containing protein 3), as previously reported [144,145].

Besides their role as pro-inflammatory mediators, mitochondria released by damaged cells can also be involved in tissue repair processes, following an injury. In particular, we recently reported that the mitochondria transfer from apoptotic endothelial or cardiac cells to MSCs constitutes a signaling messenger that triggers a cytoprotective response in the recipient MSCs, consisting of the enhanced donation of mitochondria by MSCs towards damaged cells to rescue them [119]. Interestingly, this study underlined that two DAMPs, namely reactive oxygen species (ROS) and heme, mediate the effects of damaged mitochondria in the recipient MSCs. These two DAMPS are involved at different levels of the cascade of events leading to the activation of the MSCs. First, our findings indicated that the ROS produced by damaged cells are critical regulators of the transfer of the mitochondria from the damaged cells towards MSCs, as the use of ROS scavenger abrogated both the mitochondrial transfer from the injured cells to the MSCs and the resulting MSC rescuing function [119]. The role of ROS signaling as modulator of mitochondria transfer and its associated effects have also been reported in the context of cancer, between leukemic cells and bone marrow-derived MSCs [146]. The molecular mechanisms whereby mitochondrial ROS produced by injured cells activate the cytoprotective functions of MSCs remain to be fully investigated. However, one possible mechanism is that ROS produced in excess by damaged mitochondria are sensed by MSCs as the signal triggering the degradation of dysfunctional organelles, as previously reported in other experimental settings [147,148]. The second DAMP involved in the activation of the recipient MSCs was the mitochondrial heme, released in the cytosol following the degradation of the transferred damaged mitochondria. In response to the increased level of cytosolic free heme, which has powerful pro-oxidant and toxic capacities, MSCs were found to enhance their expression of the heme oxygenase I (HO-1) enzyme to catalyze the degradation of heme, thus showing the role of HO-1 signaling in the stimulation of the pro-healing properties of MSCs [119].

## 7. Mitochondria and Microbiota: Two Sources of Metabolites for Cell Metabolism and Functions

### 7.1. Endosymbiotic Origin of the Mitochondria

Mitochondria and their host eukaryotic cells have a now well-documented endosymbiotic history showing the bacterial origin of the mitochondria. Over time, this endosymbiosis resulted in gene transfers between the mitochondrial and nuclear genomes [9,149]. As described above, this concerns the genes encoding the various subunits of the complexes of the electron transport chain, which are found in both the nucleus and the mitochondria. Many other mitochondrial proteins are encoded in the nucleus, translated in the cytoplasm, and transported to mitochondria where they exert their biological functions. Reciprocally, through the metabolites they produce, mitochondria have the capacity to exert retrograde controls on nuclear gene expression. Such is the case for alpha-ketoglutarate, 2-hydroxyglutarate, succinate, and fumarate, which regulate nuclear gene expression via the activity of DNA demethylases (Figure 3) [14].

### 7.2. Role of the Gut Microbiota in Physiology and Disease

Bacteria are actually present in mammals, in particular in their gut. The complex set of bacteria constituting the gut microbiota provides essential functions for the host mammalian body, as shown by numerous publications in this rapidly evolving area of research [150,151,152,153,154]. Its effects are not restricted to organs located near the gut, but extended to all organs as shown by liquid chromatography–tandem mass spectrometry (LC–MS/MS) of samples collected from 29 organs from either germ-free (GF) or specific-pathogens-free (SPF) mice [155]. The gut microbiota contributes to metabolically balanced physiological conditions, including the regulation of the immune system. The gut microbiota was found to alter the efficacy of immunotherapies based on the blockade of programmed cell death 1 (PD-1) or its ligand (PD-L1). These immune effects were observed for cancers whose primary sites were distant from the gut, like melanoma, non-small cell lung carcinoma (NSCLC), and renal cell carcinoma (RCC) [154]. In NSCLC and RCC patients, the presence of *Akkermansia muciniphila* in their gut microbiota was associated to a better response to PD-1 blockade [156]. Santoni and collaborators proposed that the role of *A. muciniphila* in promoting an immune response to anti PD-1 treatment could be attributed to the short-chain fatty acids (SCFAs), mainly acetate and propionate, produced by these bacteria. These SCFAs can activate the G-protein-coupled receptors (GPRCs), GPR41 and GPR43, with expected downstream effects on both cancer cell apoptosis and immune response [157].

The composition of the gut microbiota has also been recently tightly linked to malignancies. While the interaction of fecal bacteria with human colorectal cancers was expected, much less expected was the persistent association of bacteria like *Fusobacterium nucleatum* and its associated gram-negative microbiota with the colon cancer metastatic cells, at sites distant from the primary tumor. This was the case, for instance, for metastases in liver, which was associated with tumor progression [154,158]. As pointed out by the authors, the findings that the bacteria *Fusobacterium* travel with the primary tumor cells to their metastatic sites suggest that tumor microbiota might constitute an essential component of the tumor microenvironment [158].

### 7.3. Role of the Short-Chain Fatty Acids (SCFAs) Secreted by the Microbiota

Outside from physiological conditions, the gut microbiota can also either support or mitigate the metabolic functions of mammalian cells, in pathological conditions or when under metabolic stress. A lot of the effects of the microbiota, through its secreted metabolites, actually go through the functioning of their “distant cousins, the mitochondria” as recently reviewed by Agrawal and collaborators [150].

An important part of the metabolites circulating in mammals are not produced by the mitochondria-containing mammalian cells, but instead originate from the commensal bacteria found in the gut. These microbiota metabolites are mainly produced through the bacterial metabolism of dietary products and host molecules. These microbiota metabolites include lactate and the short-chain fatty acids (SCFA) acetate, propionate, and butyrate, in roughly a 3:1:1 ratio [150,152]. They provide the means for the metabolic interactions between the gut microbiota and the human host and play a key role for the overall metabolism in physiological and pathological conditions, as well as for the response to therapy treatments [150,151,152,159].

Acetate can be processed to acetyl-CoA through the enzymatic activity of the nucleocytosolic acetyl-CoA synthetase 2 (ACSS2). The overexpression of ACSS2 was observed in diverse cancers including glioblastoma and brain metastases, hepatocellular carcinoma, and breast and colorectal cancers and correlated with tumor progression both in humans and in murine models [160,161,162]. ACSS2-dependant production of Acetyl-CoA is used by the cells for lipid synthesis and acetylation of histones [161]. Therefore, as expected, high levels of acetyl-CoA lead to the increased acetylation of histones and, consequently, to the expression of genes, including those involved in cell growth. When nutrients are fully available, the acetyl-CoA comes primarily from the citrate produced from the TCA cycle in the mitochondria. However, in glioblastoma and brain metastases, the amount of the acetyl-CoA originating from acetate and feeding in the TCA cycle was substantially increased [160]. As shown by Gottlieb and collaborators [162], acetate can be processed to acetyl-CoA in conditions of low oxygen and low lipid concentrations, and thus can provide an alternative carbon source for the synthesis of fatty acids and cholesterol. In these metabolically stressed conditions, the upregulated expression of ACSS2 was responsible for cancer cell growth and survival by increasing lipid biomass. Therefore, there too, the acetate produced by the gut microbiota is expected to play a determinant role in supporting lipid biosynthesis and cancer progression.

### 7.4. Role of Other Microbiota-Secreted Metabolites

Outside from the SCFA major classes of microbiota-secreted metabolites, other metabolites produced by the gut bacteria from dietary products can influence the host cell metabolism. Such is the case for isovanillic acid 3-O-sulfate (IVAS), a metabolite produced by the microbiota upon consumption of the cyanidin 3-O-glucoside found in berries and that is detected in the blood. Houghton and coworkers showed that this compound increased the uptake of glucose and the metabolism of the differentiated human skeletal muscle myoblast line LHCN-M2 through an increased concentration and activity of the glucose transporter GLUT4, along with an activated PI3K/AKT signaling [153].

## 8. Current Therapeutic Approaches and Clinical Trials for the Treatment of Mitochondria Dysfunctions

As outlined above, diverse biological processes can account for mitochondrial dysfunctions with their load of adverse biological consequences. From the knowledge of the biological mechanisms at play, at least four types of strategies emerge for keeping control of diseases linked to mitochondrial dysfunctions:

1. directly target and repair the genes, encoded either by the mitochondrial or nuclear genomes, which are responsible for the defects;
2. use whole mitochondria to restore metabolic activities;
3. supplement the unbalanced production of metabolites from the deficient mitochondria by adding purified metabolites;
4. exploit the diversity of metabolites produced by the gut microbiota and supplement with a subset of this microbiota to provide the missing metabolites.

A number of therapeutic strategies, with their related clinical trials, have been considered in the past few years to counteract mitochondrial dysfunctions and their associated pathologies [163,164,165]. These therapies have as goals to restore the functioning of the electron transport chain, to reduce the overall oxidative stress, to supply the defective metabolites, and to enhance mitochondrial biogenesis. For most of them, however, the sought-after objective is to alleviate the symptoms rather than permanently cure the disease. The more advanced (phase III) clinical trials target genetic diseases such as mitochondrial myopathies, pyruvate dehydrogenase complex deficiency (PDCD), and Leber’s hereditary optic neuropathy (LHON) [165].

### 8.1. Gene Therapy for Both Mitochondrial and Nuclear-Encoded Proteins

One example of transient gene therapy is the expression of the wild-type and functional subunit 4 of NADH dehydrogenase (ND4), via adeno-associated virus (AAV) vectors and by intravitreal injection, in patients suffering from LHON disease in connection with the point mutation G11778A in their mitochondrial ND4 encoding gene [166]. The allotropic expression of the mitochondria-targeted ND4 in the retinal ganglion cell nuclei was followed by the import of the mitochondria-tagged ND4 protein in the mitochondria-imbedded complex I. This process restored complex I function and ATP production and preserved the visual function, as shown in both murine and rat models [167,168]. Parkinson’s disease has been linked to mitochondria dysfunction and to defective mitophagy-dependent mitochondria clearance in the dopaminergic neurons of the substantia nigra pars compacta. This is notably owing to the non-functional ubiquitin ligase Parkin and PTEN-induced putative kinase 1 (PINK1) in connection with the numerous mutations found in the corresponding PARK2 and PINK1 genes in patients with Parkinson’s disease [169,170,171]. The conditions for the viral delivery of the wild-type genes PARK2 and PINK1 in the brain have been set up in preclinical murine and rat models. However, conclusive preclinical data showing a therapeutic effect for Parkinson disease phenotypes are still lacking, thus presently precluding gene therapy clinical trials [169].

### 8.2. Therapy by Mitochondria Replacement

As detailed above, the intercellular transfers of mitochondria from MSCs to damaged recipient cells have been shown to promote beneficial wound healing effects in a wide range of pathophysiological conditions. As a result, novel mitochondria-targeted therapeutic approaches have emerged, consisting of the replacement of dysfunctional mitochondria in the injured/diseased organs through the transplantation of exogenous functional mitochondria. Mitochondrial dysfunction, leading to decreased ATP production, increased oxidative stress, apoptosis, and loss of tissue function, is the hallmark of aging and of numerous pathologies, including those caused by ischemia injury, mtDNA mutations, and metabolic disorders [172]. As a matter of fact, mitochondria transplantation has been successfully tested in several animal models for mitochondrial diseases. In particular, this therapeutic approach has been invariably shown to significantly reduce hypoxic/ischemic insult and restore tissue function following myocardial infarction [173,174,175,176], acute kidney injury [177], stroke [178,179], spinal cord injury [180,181], or optic nerve crush leading to glaucoma [182] by improving the bioenergetics and cell survival and by decreasing oxidative stress and mitochondrial DNA damages. Similarly, mitochondria transplantation has been reported to exert beneficial effects in animal models for either metabolic syndromes, including diabetic ischemic heart [183] and non-alcoholic fatty liver [184], or for neurological disorders, such as Parkinson’s disease [185,186] and schizophrenia [187]. Beyond mitochondrial disease treatment, the transplantation of exogenous mitochondrial has been evaluated in the attempt to mitigate mitochondrial dysfunction of cancer cells. This approach has been shown to attenuate the Warburg effect and to enhance the sensitivity to anti-tumoral treatments (i.e., chemotherapy or radiotherapy) in breast cancer [188] or glioma cells [189].

Overall, and similar to what is observed in the rescue of damaged cells following intercellular mitochondria transfer from MSCs, studies testing the therapeutic efficacy of mitochondria transplantation indicate that mitochondria need to be functional to exert their beneficial effects [178,182,188]. Strikingly, mitochondria used for therapeutic purposes may be of autologous, allogeneic, or xenogeneic origin, because, in all the cases, transplanted mitochondria have been reported to be non-immunogenic [178,185,186,187,188,189,190]. Although the mitochondrial transplantation procedure remains largely experimental, this technique has been successfully applied in the damaged hearts of five pediatric patients sharing ischemia-reperfusion-associated myocardial dysfunction [191].

### 8.3. Therapy by Metabolites Supplementation

A number of antioxidant molecules, such as quinone derivatives, are currently used for patients with mitochondrial diseases [165]. A water-soluble analog of coenzyme Q10, idebenone, was reported to improve the ATP production in the affected retinal ganglion cells from LHON patients and to improve the disease symptoms. Current efforts are devoted to modify these compounds so as to reduce their lipophilicity, with the goal of increasing their bioavailability. However, safety issues also need to be monitored as anti-oxidants such as N-acetylcysteine (NAC) and vitamin E are also likely to promote tumor progression [165].

Supplementation with coenzyme Q10 may also be a therapeutic option for patients suffering from heart failure, whose severity is associated with reduced levels of CoQ10. There have been numerous clinical trials testing CoQ10 supplementation for heart failure patients, notably based on the anti-oxidant capacity of CoQ10 and on the assumption that it could support the production of ATP in the defective cardiomyocytes, through the activation of the electron transport chain [192]. The Q-SYMBIO latest trial and longer (two-year end-point) to date suggested that the supplementation with CoQ10 reduced cardiovascular death, but will definitely need to be confirmed owing to the small number of patients tested [192].

Still, despite all current clinical trials, there are no FDA-approved treatments for curing mitochondrial diseases or even mitigating their effects [165].

### 8.4. Therapy by Metabolite-Producing Microbiota

Depending on the properties of the metabolites produced by specific microbiota, which can be either beneficial or harmful for humans, the corresponding microbiota can be therapeutically supplemented to the patients, or instead specifically removed with adapted antibodies. Current clinical trials actually include the supplementation of the microbiota of cancer patients undergoing strong antibiotic treatments prior to bone marrow transplantation [159]. Microbiota supplementation can be performed either by heterologous or homologous fecal microbiota transplant (FMT). The latter possibility can be used, for instance, with the microbiota of patients that can be stored during the patient microbiota-damaging treatment, such as radio- or chemo-therapy, in order to prevent further microbiota-related therapeutic side effects [159].

## 9. Concluding Remarks

While merely considered as the powerhouse of the cells for a long time, mitochondria have provoked great enthusiasm in the scientific community in this past decade. A wide number of recent investigations have recognized mitochondria as essential hubs governing cell fate, through the regulation of their bioenergetics and the production of various metabolites. On top of that, mitochondria were shown to have the capacity to translocate between cells. As a result, these fascinating organelles control multifaceted biological processes including wound healing and inflammation and their dysfunctions are associated with a broad range of diseases including inherited mitochondrial disorders and metabolic diseases such as diabetes and cancer. The full understanding of the fine metabolic mechanisms that allow mitochondria to exert their biological/signaling effects will undoubtedly need further investigations. However, the rapid scientific progress in this field highlight the key role played by the metabolites produced through the tightly interconnected ETC and TCA cycles and by mitochondrial components like DAMPs. Although additional research is needed to identify the specific metabolites/mitochondrial components responsible for the given effects of mitochondria, several therapeutic options can now be envisioned to treat mitochondrial diseases and cancer and to promote wound healing in injured/degenerative tissues, by focusing attention on whole mitochondria as well as on the metabolites/compounds they produce.

## Abbreviations

AAV	Adeno-associated virus
ACSS2	Acetyl-CoA synthetase 2
AGC	Aspartate-glutamate carrier
aKG	Alpha-ketoglutarate
AKT	Protein kinase B
ALA	Lipoic acid (1,2-dithiolane-3-pentanoic acid or thioctic acid)
ATP	Adenosine triphosphate
BAT	Brown adipose tissue
BCL-XL	B-cell lymphoma-extra large
CoQ10	Coenzyme Q10
CPEO	Chronic progressive external ophthalmoplegia
CPT1	Carnitine palmitoyltransferase 1
DAMP	Mitochondrial damage-associated molecular patterns
dGTP	Deoxyguanosine triphosphate
DHODH	Dihydroorotate dehydrogenase
DNAJA4	Dnaj heat shock protein family (Hsp40) member A4
dNTP	Deoxynucleotide triphosphate
EMT	Epithelial-to-mesenchymal transition
ERK	Extracellular signal-regulated kinase
ETC	Electron transport chain
FAD	Flavin adenine dinucleotide
FAO	Fatty acids beta-oxidation
FMT	Fecal microbiota transplant
FOXP3	Forkhead box P3
FPP	Farnesyl pyrophosphate (or diphosphate)
GAPDH	Glyceraldehyde 3-phosphate dehydrogenase
GF	Germ-free
GOT2	Glutamate-oxaloacetate transaminase
Gpx4	Glutathione peroxidase 4
HCC	Hepatocellular carcinoma
2-HG	2-hydroxyglutarate
HIF	Hypoxia-inducible factor
HLRCC	Hereditary leiomyomatosis and renal cell cancer
5hmc	5-hydroxymethylcytosine
HMGCR	HMG-CoA reductase
HO-1	Heme oxygenase- 1
Jmjc	Jumonji C
KDM	Histone lysine demethylase
KRT19	Keratin 19
KSS	Kearns–Sayre syndrome
LC–MS/MS	Liquid chromatography–tandem mass spectrometry
LDHA	Lactate dehydrogenase A
LHON	Leber’s hereditary optic neuropathy
5mc	5-methylcytosine
MCL1	Induced myeloid leukemia cell differentiation protein
MEFs	Mouse embryonic fibroblasts
MELAS	Mitochondrial encephalomyopathy with lactic acidosis and stroke-like episodes
MERRF	Myoclonic epilepsy with ragged-red fibers
MGMT	O-6-methylguanine-DNA methyltransferase
MIDD	Maternally inherited diabetes and deafness
MSC	Mesenchymal stem cell
mtDNA	Mitochondrial DNA
MTHFD	Methylenetetrahydrofolate dehydrogenase
NAC	N-acetylcysteine
NADH	Nicotinamide adenine dinucleotide
NARP	Syndrome neuropathy, ataxia, and retinitis pigmentosa
ND4	NADH dehydrogenase subunit 4
nDNA	Nuclear DNA
NOX	Nicotinamide adenine dinucleotide phosphate oxidase
NSCLC	Non-small-cell lung carcinoma
2-OG	2-oxoglutarate
OXPHOS	Oxidative phosphorylation
PARP	Poly(ADP)-ribose polymerase
PC	Pyruvate carboxylase
PD1	Programmed cell death 1
PDAC	Pancreatic ductal adenocarcinoma
PDCD	Pyruvate dehydrogenase complex deficiency
PDH	Pyruvate dehydrogenase
PDK2	Pyruvate dehydrogenase kinase 2
PDM	Peridroplet mitochondria
PFKP	Phosphofructokinase
PGK1	Phosphoglycerate kinase
PHD	Prolyl hydroxylase
PI3K	Phosphatidylinositol 3-kinase
PINK1	PTEN-induced putative kinase 1
PPP	Pentose phosphate pathway
pVHL	Von Hippel–Lindau protein
RCC	Renal cell carcinoma
RISP	Rieske iron-sulfur protein
ROS	Reactive oxygen species
SCFA	Short-chain fatty acid
SDH	Succinate dehydrogenase
SLC2A	Solute carrier family 2
SMAD	Mothers against decapentaplegic homolog 1
SNAIL	Snail family transcriptional repressor
SOD	Superoxide dismutase
SPF	Specific pathogens-free
SPOCK	SPARC (Osteonectin), Cwcv, and Kazal like domains proteoglycan
STAT	Signal transducer and activator of transcription
TAG	Triacylglyceride
TCA	Tricarboxylic acid
TET	Ten-eleven-translocation
TGFβ	Transforming growth factor beta
TNT	Tunneling nanotube
Treg	Regulatory T cell
VEGF	Vascular endothelial growth factor
